# Sacral Erector Spinae Plane Block As the Main Anesthetic Method for Parasacral Reconstructive Surgeries: A Single-Center Retrospective Cohort Feasibility Study

**DOI:** 10.7759/cureus.37347

**Published:** 2023-04-09

**Authors:** Murat Unal, Hakan Baydar, Serkan Guler, Ayhan Sonmez, Murat Gumus, Serkan Tulgar

**Affiliations:** 1 Department of Anesthesiology and Reanimation, Samsun University Faculty of Medicine, Samsun Training and Research Hospital, Samsun, TUR; 2 Department of Plastic, Reconstructive, and Aesthetic Surgery, Samsun University Faculty of Medicine, Samsun Training and Research Hospital, Samsun, TUR; 3 Department of Plastic, Reconstructive and Aesthetic Surgery, Samsun University Faculty of Medicine, Samsun Training and Research Hospital, Samsun, TUR; 4 Department of Anesthesiology, Samsun University Faculty of Medicine, Samsun, TUR

**Keywords:** ultrasound-guided intervention, sacral pressure ulcer, mechanism of action erector spinae plane block, sacral erector spinae plane block, ultrasound-guided regional anesthesia

## Abstract

Study objective

Sacral erector spinae plane block (ESPB) is a regional anesthesia technique defined for the blockade of the posterior branches of the sacral nerves. In this study, we aimed to retrospectively evaluate our sacral ESPB applications as an anesthetic method in patients who underwent parasacral and gluteal reconstructive surgery.

Methodology

The design of our study is a retrospective cohort feasibility study. This study was conducted at a tertiary university hospital, and patient files and electronic data systems were used to obtain data for analysis. In total, the data of 10 patients who underwent parasacral or gluteal reconstructive surgery were evaluated.

Main results

During reconstructive procedures for sacral pressure ulcers and lesions in the gluteal region, the sacral ESP block was utilized. Small doses of perioperative analgesics/anesthetics were required, but moderate or deep sedation or conversion to general anesthesia was not required.

Conclusion

Sacral ESP block is a viable regional anesthetic technique in reconstructive surgeries of the parasacral and gluteal regions.

## Introduction

An ultrasound-guided erector spinae plane block (ESPB) is a popular interfascial plane block described by Forero et al. in 2016 for the treatment of thoracic neuropathic pain [1]. Although initially described for chronic pain, its use as part of a multimodal analgesia plan for perioperative and postoperative analgesia has been frequently reported [1-3].

Although ESPB was originally defined as the application of local anesthetic to the interfascial plane between the erector spina muscle group and the transverse process of the thoracic spine for the indication of relief of thoracic neuropathic pain at the thoracic (T4-5) level, it was later used for various indications to relieve acute and chronic pain. In addition, its applications at the cervical, thoracic, and lumbar vertebral levels have also been described [2,4].

Application of ESPB in the sacral region has been described by Tulgar et al. [5] to provide sensory blockade in the parasacral region by blocking the posterior branches of the sacral nerves, and its use has been reported for many indications ranging from anorectal surgery to orthopedic surgery [6-9].

The aim of this retrospective study was to evaluate the feasibility of this technique as a main anesthetic method in patients undergoing parasacral reconstructive surgery in which sacral ESPB was selected as a component of anesthesia management.

## Materials and methods

Study design

This study was designed as a retrospective analysis of collected data. Local ethics committee approval was taken (SÜKAEK-2022/7/3). Data were collected from hospital automation systems (FONET v4.23.1.1, Turkey) for patients who underwent parasacral reconstructive surgery between September 2021 to September 2022 in Samsun University Education and Research Hospital. Data of the American Society of Anesthesiology (ASA) I-IV adult patients scheduled for elective parasacral reconstructive surgery were collected. Patients who underwent sacral ESPB as the main anesthesia method or as part of the anesthesia plan were included in the study. Patients who underwent surgery under general or neuraxial anesthesia or infiltration anesthesia with sedoanalgesia were excluded. In our daily practice, all patients were provided with detailed information about the anesthesia techniques and possible complications, and their written consent was obtained. The surgical procedures described in this article were performed by two plastic and reconstructive surgeons.

Data collection

Patient demographic data such as age, gender, weight, height, and the American Society of Anesthesiology (ASA) physiological classes were noted. Surgery and anesthesia-related descriptive data were also collected, which included the type and duration of surgery, the level and side of block(s), the dose and volume of local anesthetic (LA) used, sensorial block onset time, the need for additional anesthetic/analgesic medication, and the duration of the first analgesic requirement.

Anesthesia protocol

After all the patients were taken to the operating room, routine anesthesia monitoring was performed, and nasal O_^2^_ was administered at a flow of 2-4 lt/min. Patients were premedicated with intravenous 0.01-0.02mg/kg midazolam and 0.25 mcg/kg fentanyl (or 0.15mg/kg ketamine). After confirming the continuity of hemodynamic stability after sedoanalgesia, the patients were placed in the prone position.

All blocks were performed under aseptic conditions with the in-plane technique and a low-frequency convex transducer (3-5 MHz, Esaote MyLab™30 Gold, Genoa, Italy). Needle orientation was from lateral to medial in the sacral erector spinae plane (ESP) and from caudal to cephalad in the superior cluneal nerve block. A peripheral nerve block needle (Vygon Echoplex, 85 mm, 21 G, Ecouen, France) was used in the blocks. All blocks were performed by an experienced anesthesiologist in sacral ESPB.

Sacral ESPBs were performed at the S1-S2 retrolaminar area level. First, the ultrasonography (USG) transducer was positioned in the lumbar region with the spinous process in the center of the image. Afterward, the sacral spinous processes were observed by sliding the probe caudally toward the sacrum. By shifting the transducer laterally in the transverse plane, the intermediate sacral crest, the multifidus muscle just above it, and the skin and subcutaneous tissues were sonographically identified.

The plane was confirmed by injecting a few mL of saline into the plane deep in the multifidus muscle, and then LA was injected into this plane, and craniocaudal and mediolateral LA spread was observed. In our clinical practice, a mixture of LA with a concentration of 0.25% bupivacaine and 0.5% lidocaine was used for surgical anesthesia blocks.

Although sacral ESPB was applied at S2 level in general, S1 level was preferred in cases such as skin infection. The pinprick test was performed intermittently. Target-specific additional regional anesthesia techniques were planned for patients who were determined to have insufficient sensory block coverage for the planned surgical field by the 45^th^ minute. When adequate cutaneous blockade was achieved, the surgical procedure was initiated. Although cutaneous block was considered adequate, 10 mg of ketamine or 25 mcg of fentanyl was administered to patients reporting mild to moderate pain in the perioperative period. Which additional analgesic to use was decided by the anesthetist according to the clinical conditions of the patient.

There is a routine postoperative analgesia plan for patients undergoing parasacral constructive surgery, and this includes intravenous administration of paracetamol every eight hours with the first dose at the end of the operation. In the ward, different non-steroidal anti-inflammatory drugs (NSAIDs) are administered as rescue analgesics - according to the medical condition of the patients - for those reporting numeric rating scale (NRS) scores of four and above.

Statistics

Data were analyzed using the Statistical Package for the Social Sciences (SPSS) version 16 (IBM Inc., Armonk, New York) package program. Continuous quantitative data were presented as number, mean ± standard deviation.

## Results

It was determined that a total of 37 patients underwent sacral and gluteal region reconstructive surgery. Sacral ESPB was applied to 15 of them. Five patients who underwent sacral ESPB for postoperative analgesia were excluded from the study. Eight male and two female patients aged between 30 and 87 (52.2±21.48) years were included in the study. The mean BMI of the patients was 22.83±2.53 kg/m^2^. Demographic data of the patients, such as ASA classes, additional systemic diseasesm and comorbiditiesm are presented in Table 1.

**Table 1:** Demographic data, surgery types, regional anesthesia features, and analgesic requirements ASA - American Society of Anesthesiology, ESP - erector spinae plane, LA - local anesthetic, DM - diabetes mellitus, HT - hypertension, CF - cardiac failure, CVD - cerebrovascular disease, SCNB - superior cluneal nerve block, NA - not applicable

Case number	Age/ gender	BMI kg/m^2^	ASA/ comorbidities	Diagnosis/planned surgery	Sacral ESP side LA volume	Additional block LA volume (mL)	Block onset time (minutes)	Surgery duration (minutes)	Perioperative analgesic requirement	First analgesic requirement time after surgery(hours)
1	36/M	19.37	1/NA	Sacral pressure ulcer/ rotational flap	Bilateral 30/30 mL	NA	30	30	10mg ketamine	9
2	33/F	20.57	1/NA	Gluteal abscess/ debridement, drainage	Right 30 mL	SCNB 10 ml	55	45	50mcg fentanyl	11
3	87/M	21.11	3/DM, HT	Sacral pressure ulcer/ debridement	Right 30ml	NA	35	21	10mg ketamine	9
4	67/M	23.84	2/DM, HT	Sacral pressure ulcer/ debridement	Bilateral 20/20 mL	NA	25	47	25mcg fentanyl	10
5	45/M	24.82	2/HT	Sacral pressure ulcer/ debridement	Bilateral 30/30 mL	NA	30	40	25mcg fentanyl	10
6	49/M	24.97	3/HT, paraplegia	Pressure ulcer/ rotational flap	Bilateral 30/30 mL	NA	32	39	NA	10,5
7	30/M	22.34	3/transverse myelitis	Sacral pressure ulcer/ debridement	Bilateral 30/30ml	NA	40	60	25mcg fentanyl	8
8	83/F	25.53	3/DM, CF, CVD	Sacral pressure ulcer/ rotational flap	Bilateral 20/20ml	NA	25	42	50mcg fentanyl	8,5
9	62/M	26,15	3/DM, HT	Sacral pressure ulcer/ debridement	Bilateral 20/20ml	NA	30	35	10mg ketamine	11
10	30/M	19,69	3/paraplegia	Sacral pressure ulcer/ debridement	Bilateral 30/30 mL	NA	30	45	NA	13

When the patients were evaluated in terms of the sides of the block, it was bilateral in eight and unilateral in two cases. Of the patients with sacral pressure ulcers, three patients underwent rotational flap surgery, while six patients underwent extensive wound debridement. Abscess drainage and wide excision were performed in a patient who developed a deep abscess after gluteal intramuscular injection, for which an additional superior cluneal nerve block was added for anesthesia. 

**Figure 1:**
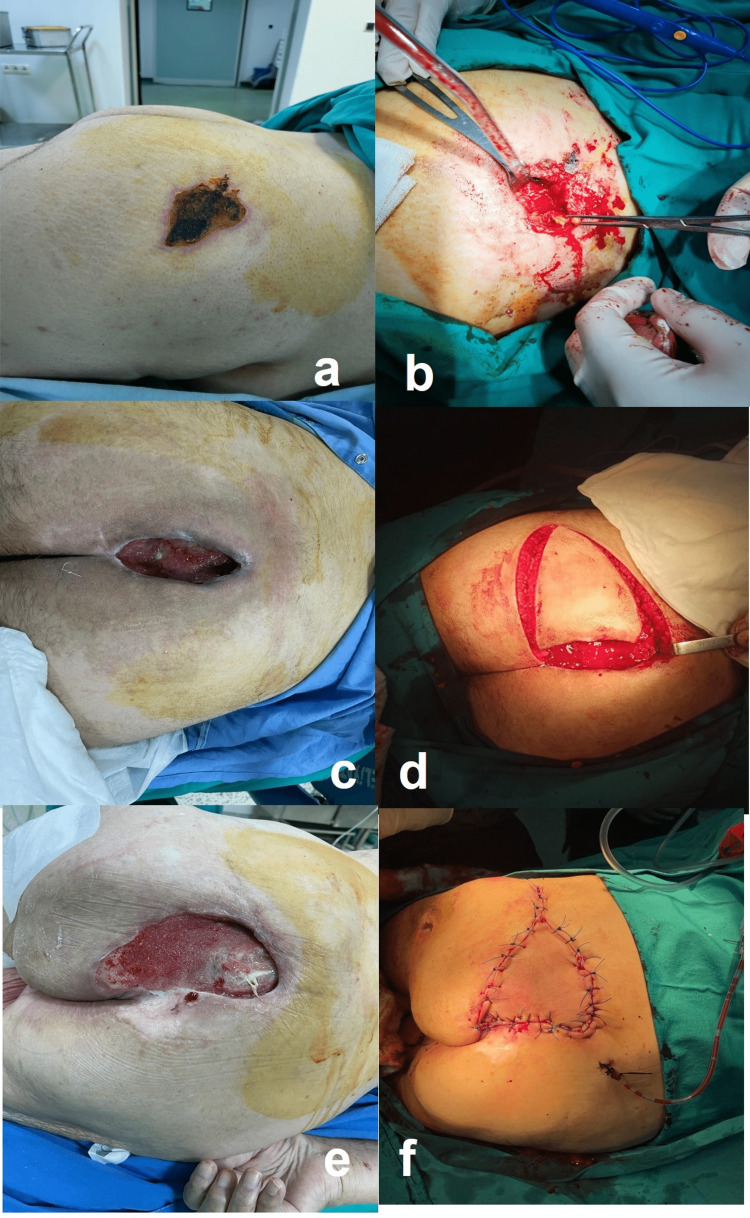
Demonstration of preoperative and perioperative images of case two (a, b), case one (c, d), and case six (e, f)

All but one patient complained of mild to moderate pain at least once during surgical manipulations and received low doses of ketamine and/or fentanyl. The administered doses are presented in Table 1 in detail. In the postoperative follow-up, the time to first analgesia requirement was determined to be 10±1.47 hours. In addition, examples of surgical procedures performed are presented in Figure 1.

## Discussion

In this retrospective study, we observed that sacral ESPB may be a feasible option as a main anesthetic method when applied alone or in combination with other plan blocks in parasacral and gluteal region reconstructive surgeries.

Cutaneous innervation of the parasacral and gluteal regions is generally considered to be provided by the posterior branches of the sacral nerves [5]. However, when the innervation of the lumbosacral region is examined in detail, it will be seen that it is a very intricate innervation network. The cutaneous innervation of the parasacral area is provided by the middle cluneal nerve (S1-S3 posterior branches), while the lateral cutaneous branches of the subcostal and iliohypogastric nerves in the lateral gluteal region and the superior cluneal nerve in the middle gluteal region. The superior cluneal nerves are formed by the union of the lateral branches of the dorsal rami of the L1-L3 nerves and pass over the crista iliaca and innervate the upper part of the buttock. In addition, the inferior cluneal nerves, a branch of the posterior femoral cutaneous nerve, innervate the inferior gluteal region [10]. 

We applied a sacral ESP block for reconstructive surgeries in the parasacral region, and a superior cluneal nerve block was added for incisions involving regions far from the midline. The superior cluneal nerve provides sensory innervation to the upper lateral gluteal region, which is why we added it as a complementary block. If surgeries involving inferior gluteal area incisions were required, a posterior femoral cutaneous nerve block would be logical to use as a complementary block [11]. In addition, combinations including blocks such as lateral femoral cutaneous nerve block and transversalis fascia plane block (for lateral cutaneous branches of T12-L1 nerves) may be preferred depending on the extent of the surgical incision [12].

Although the sacral ESP block was originally defined for the blockade of the posterior branches of the sacral nerves, studies have shown that when the sacral ESP block is applied with a high volume or modified, the injectate can spread from the sacral foramina to the nerve roots or to the epidural space via the transforaminal route [13,14]. Indeed, reported anecdotal cases support such disseminations [6]. 

Our study has some limitations. First of all, our retrospective design increases the susceptibility to bias. In addition, although we performed a sensory analysis of the area planned for surgery with the pinprick test after block applications, we had patients who complained of mild-to-moderate pain perioperatively -due to possible surgical manipulation-. If we had used the infusion of dexmedetomidine or a similar agent for sedoanalgesia and compliance with surgery, we could have provided more comfortable anesthesia by preventing these pains. Nine out of 10 patients had pressure ulcers, and it is known that one of the common causes of pressure ulcers in the sacral and ischial region is sensory loss. We did not perform a detailed sensory analysis before the block, but although sedoanalgesia was applied in all patients, a response to pain was observed during the application of sacral ESPB during skin penetration or bone contact. However, these will need to be analyzed in detail in a prospective study to be conducted.

## Conclusions

Our study demonstrated that sacral ESP block is a feasible technique in anesthesia management in reconstructive surgeries of the parasacral and gluteal regions, and it is an option that clinicians should keep in mind as an alternative technique in high-risk patients. The mechanism of sacral ESP block should be elucidated with further anatomical and clinical studies.
